# Homœopathy: An Examination of Its Doctrines and Evidences

**Published:** 1852-03

**Authors:** 


### ARTICEL II.

*Fisks' Fund Prize Dessertation of the Rhode Island Medical Society. Homœopathy: An examination of its Doctrines and Evidences*: By Worthington Hooker, M. D., author of "Physician and Patient," and "Medical Delusion." New York: Charles Scribner, 145 Nassau St., 1851. Pages, 146, Duodecimo. (From the Publisher through A. H. & C. Burley of Chicago.

In this work the author does not merely aim at a refutation of the doctrines of the Homœopathists, but endeavors to show up the general principle upon which fallacy in all special systems originates.

In reference to the mode of discussing the subject of Homœopathy, the author remarks in his introduction—

"Homœopathy is so absurd, that it seems almost a waste of time and effort to go through a formal refutation of it. And so it would be, were its refutation not made necessary, from its adoption by so many of the intelligent and influential among the non-medical portion of the community. Such persons, I trust, will find, on reading this essay, that their belief in the system of Hahnemann has been formed without a real understanding of its merits. And I flatter myself that those of them who will give me a candid hearing, will be induced to abandon such a combinatinn of falsities and inconsistencies as this system presents.

Homœopathists complain that physicians ridicule their doctrines, and very gravely say, that the system of the "sage of Cœthen," is not to be put down by a laugh. But when things are exceedingly laughable, it is a little unreasonable to demand of us an imperturable gravity. When Homœopathy conjures up its ridiculous fantasies to play before us like so many harlequins, it is hard to be denied the privilege of laughing at them. As to the alleged impropriety of ridicule in the discussion of the merits of this system, it may be remarked, that it cannot be improper if it only be used fairly; and if a little pleasantry suffice to demolish an error, it surely is an unnecessary waste of power to attack it with strong and sober argument. It were folly to deal sturdy blows at bubbles which can be dissolved by the slightest touch."

The work is every way readable, argumentative, amusing, and convincing, and needs but to be placed in the hands of intelligent men to give the little pill system its just deserts, for surely no one would tolerate such humbuggery after so full and what appears to us, so fair an exposure.

The author shows up very fully the errors and follies of the modes by which the Homœopathists detect the therapeutical applicability of remedies, which our readers all know to be by observing the symptoms that are produced by their use in health. Certainly the results of such a trial, with any medicine diluted as is recommended by the great exemplar, would be fanciful enough for the most spiritual and visionary deciple that Hahnemann ever had.

In reference to dilutions we quote one of his arithmetical cal culations with the note he has appended to it.

"Let us try and get at the minuteness of this dilution. Let us see what quantities of liquid would be required for the successive dilutions, if instead of throwing away ninety-nine parts out of every hundred, the whole is retained. For the first dilution one hundred drops of alcohol would be used. For the second it would take ten thousand drops, or about a pint. For the third it would take one hundred pints. For the fourth ten thousand pints. And now it mounts up rapidly at each dilution. For the ninth dilution it would take ten billion of gallons, which, according to computation of Dr. Panvani, equals the quantity of water in the Lake Agnano, which is twelve miles in circumference. For the twelfth dilution a million of such lakes would be required, or as it is reckoned by Dr. Post of New York, (from whom I shall take the liberty to borrow the remaining calculations rather than attempt them myself,) it would reque five hundred lakes as large as Lake Superior.

The fifteenth dilution would require a quantity of alcohol greater in bulk than the earth. The eighteenth would require a quantity greater than the volume of the sun. And the thirtieth, the one which Hahnemann insists upon as being the best for common use, would take a quantity of alcohol exceeding the volume of a quadrillion of suns."*

The work is printed in good style, and we should think, would meet with an extensive sale. Physicians would do well to procure it and loan it to their intelligent friends, who may have been deceived to a faith in the small doses. If they are not convinced by it, they may be given over as incorrigible.

---

* The following jeu d'esprit, appeared in a newspaper, so far from being a caricature, as the reader will see, falls far short of the absurdity of Homœopathy. It is a prescription for a Homœopathic rum cordial.

Take a little rum,

The less you take the better;

Pour it in the lakes

Of Wener and of Wetter.

Dip a spoonful out,

Mind you don't get groggy,

Pour it in the lake

Winnipissiogee.

Stir the mixture well,

Lest it prove inferior,

Then put half a drop

Into Lake Superior.

Every other day

Take a drop in water,

You'll be better soon,

Or at least you ought to.

Attenuated as the dilution here described is, it falls very far short of the higher attenuations of Homœopathy, and especially that which is in so common use, the thirtieth dilution.

